# The Healthy Home Laboratory: Implementing Smart Technology to Promote Aging in Place

**DOI:** 10.1093/geroni/igaf122.1881

**Published:** 2025-12-31

**Authors:** Pamela Toto, Steven Handler

## Abstract

Older adults prefer to remain in their homes as they age. However, according to U.S. Census Bureau, only about 10% of homes are “age-ready.” Many older homes were not designed to support age-related changes and pose unique barriers for aging-in-place. To tackle these challenges, the University of Pittsburgh launched the Healthy Home Laboratory (HHL). HHL is a university-community laboratory established in a 107-year-old home that typifies older housing. An interprofessional team and community partners collaborate in this space to design, develop, evaluate and deploy smart technology solutions for aging-in-place. Representative of real-world challenges in homes, HHL reduces the knowledge-to-practice gap between science and everyday living and accelerates the implementation of solutions that are acceptable, sustainable and effective. This symposium provides an overview of the HHL and showcases smart technologies being implemented through the HHL to complement traditional home modifications for community impact. An introduction to Home Safe and Smart will share facilitators and barriers to integrating smart technology into a person-centered intervention offered through a health plan. Presentations of work funded by the Department of Housing and Urban Development will share results of efforts to implement a smart technology air quality assessment program within existing home and community-based services and work-in-progress to implement a smart technology fall prevention package and medication adherence solutions into low-income senior housing apartments. The final presentation will share results of a pilot supported by the Veterans Affairs to develop a technology preparedness assessment to help Veterans access care through telehealth.

